# Correspondence—Original

**Published:** 1856-04

**Authors:** J. Harris

**Affiliations:** Salem, Ohio


### Correspondence.

### A DENTAL EXCURSION.

Mr. Editor:—Having just returned from a very pleasant and interesting excursion amongst some of the most talented of our profession in Philadelphia; also having visited Professor Harris of Baltimore, I thought possibly I might impart some hints as connected with my trip, that would not be entirely void of interest to your readers.

To commence with the Dental College, the lecture rooms of which, are in the upper stories of the spacious establishment of Jones, White & McCurdy, 116 Arch street, I will say, that I found everything apparently in a prosperous condition. The class was mostly composed of talented looking young men, whose attention to the instructions given, gave evidence of a commendable interest in the subjects respectively brought before them. Of the lectures I listened to, I shall only refer to one by Prof. Arthur, in which he was discussing the subject of filling cavities in which the nervous pulp was shielded by a thin lamina of osseous substance. He thinks that in such cases, it is better not to remove such portions of dentine, even though considerably discolored, than to risk the exposure of the nerve. And where we suspect this portion of osseous covering to be so frail as not to support the necessary amount of pressure required for condensing the gold, he recommends capping or plating as a protection. He referred to cases in which the lamina of dentine thus protected, although much discolored, at the time of the operation of filling, had assumed a healthy condition afterwards, and had proved very successful.

Prof. J. D. White was just recovering from an attack of Quinzy, which had prevented him from delivering two lectures which I intended to hear. I called on him at his office however, where I was most pleasantly and profitably entertained. I brought with me a very fine portrait of the Professor, which I would be very happy to show to any of my Western brethren who may wish to see it. Through the kindness of Dr. Edward Townsend, for whom as a gentleman, and a member of our profession, I am under many obligations, both during my present excursion, and on a former occasion. I had an introduction to his brother, ex-Prof. Elisha Townsend, who is by many considered the "crack" dentist of Philadelphia. If the style of his equipments, the elegance, taste, and even splendor of his office and other apartments are any criterion, or if the prices which he receives for his operations are taken into consideration, we might very reasonably "book" him as such. I witnessed some very fine operations in the offices of him and his brother Edward. I called on Dr. Beale, he says he has suffered much, which his pale look goes to confirm. His practice from which he was so precipitably removed seems to be returning again. He says he does not now administer ether to man, woman, or child without the presence of a third person. I have felt quite satisfied ever since reading the testimony in his case, that he ought not to have been convicted. That the testimony was insufficient, and that in all probability he was entirely innocent of the charges preferred against him, my present interview went strongly to confirm.

It is a tribute, but justly due to the firm of Jones, White & McCurdy to say, that their treatment of me and their numerous customers whilst I was there, was of the most gentlemanly character, and that their laudable ambition to keep decidedly in the front rank, in the manufacture of teeth and other dental stock, is being crowned with the most enviable success.

In Professor Harris of Baltimore, I found a large portly looking gentleman 6 feet 2 inches high, and weighing 208fbs. His dignified countenance seemed but the index of the generous sentiments which fell from his lips during my short visit to him. He appears to be one of the hard workers in our profession; and as I doubt not has done more for the elevation of Dental surgery than any other man. Long may he live to be an ornament to the profession of his choice, and may he reap a rich reward.

From Baltimore I passed on to Washington, intending to call on Dr. Maynard, but he had retired from practice. And as it was my first visit to "the city of magnificent distances," I found so much to enlist my attention that I did not form any acquaintance among the dentists of that place.

I ascertained that the dentists generally whom I visited, were operators in full practice, and that they considered out door exercise very conducive to their health and comfort; and of the different kinds, exercise on horseback found most favor amongst them. Elisha Townsend keeps three elegant nags for the use of himself and Mrs. Townsend, a very intellectual lady, with whom I had the honor of dining with at her own table. They mostly keep at least one good horse, as a necessary appendage to the fixtures of their respective establishments. To the propriety of the adoption of this item, I presume we will all most cheerfully subscribe.

Now although my trip cost me time and money, for I was gone about a month, it paid well. I saw much that was interesting and very instructive, I extended my acquaintance, and I feel refreshed. I feel determined to perform the various operations of Dental surgery as much better than ever before as possible, and to do what I can for its elevation as a profession. J. HARRIS.

*Salem, Ohio*, Feb., 1856.

